# Marine Nutraceuticals: Prospects and Perspectives. By Se-Kwon Kim, CRC Press, 2013; 464 Pages. Price £108.00, ISBN 978-1-4665-1351-8

**DOI:** 10.3390/md11041300

**Published:** 2013-04-17

**Authors:** Shu-Kun Lin

**Affiliations:** MDPI AG, Kandererstrasse 25, CH-4057 Basel, Switzerland; E-Mail: lin@mdpi.com

The following paragraphs are reproduced from the publisher’s website [1].

There is a great deal of consumer interest in natural bioactive substances due to their health benefits. Offering the potential to provide valuable nutraceuticals and functional food ingredients, marine-derived compounds are an abundant source of nutritionally and pharmacologically active agents, with both chemical diversity and complexity. Functional ingredients derived from marine algae, invertebrates, vertebrates, and microorganisms can help fill the need for novel bioactives to treat chronic conditions such as cancer, microbial infections, and inflammatory processes.

With contributions from an international group of experts, *Marine Nutraceuticals: Prospects and Perspectives* provides a comprehensive account of marine-derived nutraceuticals and their potential health benefits. These include antioxidant, anticancer, antiviral, anticoagulant, antidiabetic, antiallergic, anti-inflammatory, antihypertensive, antibacterial, and radioprotective properties. The book focuses on various types of marine-derived compounds—such as secondary metabolites like phlorotannins and fucoxanthin, carotenoid pigments, chito-oligosaccharide derivatives from chitin and chitosan, bioactive peptides, and polysaccharides—presenting an overview of their nutraceutical activities.

Chapters address neuroprotecive properties of seaweeds, bioactive compounds in abalone, marine products and autoimmune disease, chitosan for weight management, anticancer actions of omega-3 fatty acids, chitosan in dentistry, and much more. The book discusses the sources, isolation and purification, chemistry, functional interactions, applications, and industrial perspectives of marine-derived nutraceuticals. The inaugural book in the new CRC Press series, Nutraceuticals: Basic Research/Clinical Applications, it provides a state-of-the-art reference for all readers interested in this growing field—a rich source for new compounds with promising uses in the nutraceutical, medicinal, and functional food industries.

## Table of Contents

Series PrefaceixPrefacexiiiEditorxvContributorsxvii**1 Marine-Derived Nutraceuticals: Trends and Prospects**Se-Kwon Kim and Isuru Wijesekara1**2 Nutritional Value of Sea Lettuces**Se-Kwon Kim and Ratih Pangestuti5**3 Prospects and Potential Applications of Seaweeds as Neuroprotective Agents**Se-Kwon Kim and Ratih Pangestuti17**4 Chitosan-Based Biomaterials against Diabetes and Related Complications**Se-Kwon Kim and Fatih Karadeniz33**5 Nutraceutical Benefits of Marine Sterols Derivatives**Se-Kwon Kim and Quang Van Ta45**6 Nutritional Value, Bioactive Compounds, and Health-Promoting Properties of Abalone**Mahanama De Zoysa57**7 Marine Biopolymers in Asian Nutraceuticals**Ngo Dang Nghia and Se-Kwon Kim71**8 Marine Natural Antihypertensive Peptides from *Styela clava* Having Multifunctions of ACE Inhibition and NO Production in Endothelial Cells**Seok-Chun Ko, Se-Kwon Kim, and You-Jin Jeon87**9 Beneficial Effects of Marine Natural Products on Autoimmune Diseases**Mi Eun Kim, Jun Sik Lee, Se-Kwon Kim, and Won-Kyo Jung99**10 Extraction of Nutraceuticals from Shrimp By-Products**Trang Si Trung and Willem Frans Stevens115**11 Fucoidan: A Potential Ingredient of Marine Nutraceuticals**Se-Kwon Kim, Thanh-Sang Vo, and Dai-Hung Ngo131**12 Chitosan for Body Weight Management: Current Issues and Future Directions**Soon Kong Yong and Tin Wui Wong151**13 Prospects of Indonesian Uncultivated Macroalgae for Anticancer Nutraceuticals**Hari Eko Irianto and Ariyanti Suhita Dewi169**14 Active Ingredients from Marine Microorganisms for Modern Nutraceuticals**Se-Kwon Kim and Pradeep Dewapriya187**15 Potent Anticancer Actions of Omega-3 Polyunsaturated Fatty Acids of Marine Nutraceuticals**Kaipeng Jing and Kyu Lim199**16 Chitosan Application in Dentistry**Yoshihiko Hayashi, Kajiro Yanagiguchi, Zenya Koyama, Takeshi Ikeda, and Shizuka Yamada233**17 Edible Marine Invertebrates: A Promising Source of Nutraceuticals**Se-Kwon Kim and S.W.A. Himaya243**18 Chitosan and Its Derivatives: Potential Use as Nutraceuticals**Jae-Young Je and Se-Kwon Kim257**19 Marine Sulfated Polysaccharides with Unusual Anticoagulant Action through an Additional Unrelated-Natural Inhibitors Mechanism**Bianca F. Glauser, Paulo A.S. Mourão, and Vitor H. Pomin267**20 Enzymatic Production of *N*-Acetyl-d-Glucosamine Using the Enzyme from the Liver of Squid**Masahiro Matsumiya301**21 High-Density Chitin–Chitosan Production and Beneficial in Health**Siswa Setyahadi313**22 Antioxidant Effects of Marine Food-Derived Functional Ingredients**Se-Kwon Kim, Dai-Hung Ngo, and Thanh-Sang Vo329**23 Biological and Biomedical Applications of Marine Nutraceuticals**Janak K. Vidanarachchi, Maheshika S. Kurukulasuriya, and W.M.N.M. Wijesundara345**24 Fucoidans from Marine Brown Macroalgae: Isolation, Identification, and Potential Biological Activities**Yasantha Athukorala and Yvonne V. Yuan393Index437

*Editor’s Note*: The brief summary and the contents of the books are reported as provided by the author or the publishers. Authors and publishers are encouraged to send review copies of their recent books of potential interest to readers of *Mar. Drugs* to the Publisher (Dr. Shu-Kun Lin, Multidisciplinary Digital Publishing Institute (MDPI), Kandererstrasse 25, CH-4057 Basel, Switzerland. Tel.: +41-61-683-77-34; Fax: +41-61-302-89-18; E-Mail: lin@mdpi.com). Some books will be offered to the scholarly community for the purpose of preparing full-length reviews.
